# The Effect of ‘Hydraulic‐Visual’ Interaction on the Intensity of Fish Schooling in the Upstream Movement of Juvenile Silver Carp

**DOI:** 10.1002/ece3.73659

**Published:** 2026-05-14

**Authors:** Ding Haoyu, Lin Chenyu, Wu Yujiao, Yang Zijing, Shi Xiaotao, Liang Zezhang, Rong Guiwen, Xu Jiawei, Luo Jia, Yu Lixiong

**Affiliations:** ^1^ Hubei International Science and Technology Cooperation Base of Fish Passage China Three Gorges University Yichang China; ^2^ College of Hydraulic and Environmental Engineering China Three Gorges University Yichang China; ^3^ Jingshan Water Conservancy Development Center Jingshan Water Conservancy and Lakes Bureau Jingmen China; ^4^ College of Earth and Environment Anhui University of Science and Technology Huainan China; ^5^ Pearl River Water Resources Research Institute Pearl River Water Resources Commission Guangzhou China; ^6^ Key Laboratory of the Pearl River Estuary Regulation and Protection of Ministry of Water Resources Guangzhou China; ^7^ Yangtze River Fisheries Research Institute Chinese Academy of Fishery Science; National Agricultural Science Observing and Experimental Station of Chongqing Wuhan China

**Keywords:** bighead carp, fish passage facilities, fish schooling, hydraulic stimuli, swimming energy expenditure, visual guidance

## Abstract

Understanding how hydraulic and visual factors jointly regulate fish schooling is critical for improving fish passage performance. Using juvenile silver carp ( *Hypophthalmichthys molitrix* ) as a model organism, we investigated how schooling intensity responds to the combined effects of flow velocity, turbulence kinetic energy (TKE) and visual guidance cues from obstacles. The experiment was conducted in a controlled flume with three flow conditions at a fixed fish group size (five individuals). By integrating simulated flow fields with the spatial distribution of fish schooling intensity and swimming energy expenditure, relationships among flow characteristics, energetic demand and schooling behaviour during fish upstream movement were quantitatively analysed. We concluded that (1) increasing flow velocity constrained the fish's lateral exploration and prompted them to ascend predominantly along the main flow current; (2) in relatively mild flow conditions, fish schooling intensity was barely affected by TKE, whereas in obstacle areas where high velocity co‐occurred with low turbulence, they preferred to select high‐velocity paths as efficient ascending passages near obstacles; (3) increase in short‐term or cumulative energy expenditure alone was not sufficient to drive an increase in fish schooling, whereas the surge in energy requirements under conditions of high‐velocity barriers correlated with enhanced fish schooling intensity; (4) after passing velocity barriers, fish adopted a short‐burst acceleration strategy in low‐velocity, low‐turbulence regions upstream of obstacles to avoid reforming dense schools, indicating an active tendency to minimise prolonged energy costs when possible; and (5) obstacles provided salient visual cues that enabled fish to avoid collisions and adjust their swimming direction during rapid upstream movement. Overall, these findings demonstrate that schooling intensity is a dynamic behavioural adjustment shaped by the interplay between hydraulic conditions, associated energetic costs and visual information. The results provide mechanistic insight into collective upstream migration and offer practical guidance for the hydraulic and structural optimisation of fish passages.

## Graphical abstract

This study used juvenile silver carp as model organisms to investigate the combined effects of flow velocity, turbulence kinetic energy and visual cues from obstacles on fish schooling intensity. The experiments demonstrated that schooling intensity is a dynamic behavioural adjustment shaped by the integration of hydraulic constraints, energetic demands and visual information during upstream migration. These findings provide a theoretical basis and practical guidance for elucidating the mechanisms of collective fish migration and for optimising the hydraulic and structural design of fish passages.

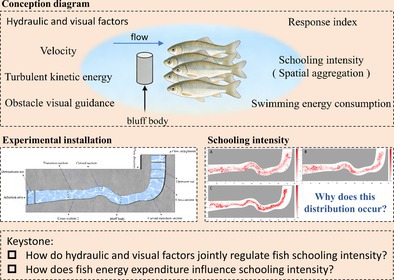

## 1 Introduction

Hydropower development plays an essential role in supporting societal progress; however, the dams and diversion structures associated with these projects often disrupt the natural migratory pathways of fish, thereby compromising the ecological integrity and biodiversity of aquatic systems (Morita and Yamamoto 2002; Qiuwen et al. 2020). To mitigate these impacts, a variety of fish passage facilities have been constructed to re‐establish migration corridors and restore river connectivity (Xingyong and Jun 2005). Considerable research effort has since been devoted to improving the operational performance of such facilities, encompassing investigations into fishway hydraulics, fish behavioural responses and structural optimisation strategies (Baoligao et al. 2011; Silva et al. 2018). Furthermore, it has been widely recognised that many fish species undertake upstream migration in schools, and that the collective behaviour within these groups exerts a pronounced influence on their passage performance (Xie et al. 2025; Nyqvist et al. 2024; Crawford et al. 2025). Studies conducted in artificial fish passage have demonstrated that fish schools exhibit markedly higher passage success rates than solitary individuals (74% vs. 48%) (Lemasson et al. 2014). Schooling behaviour is intrinsically linked to individual energy expenditure, cooperative sensing, navigation strategies and physiological stress responses (Humston et al. 2000; Okasaki, Keefer, et al. 2020; Okasaki, Keefe, et al. 2020; Mozzi, Manes, et al. 2024; Schumann et al. 2023), influencing processes, such as path selection, swimming endurance and avoidance responses during movement. Collectively, these factors shape the efficiency with which fish utilise anthropogenic migration facilities.

As a fundamental form of fish behaviour, schooling is shaped by a suite of environmental factors, among which flow conditions are a key determinant of fish locomotion (Liang et al. 2012), influencing swimming patterns, hydraulic parameter preferences and energy expenditure. During upstream migration, fish typically alternate between burst swimming and steady swimming, dynamically adjusting their propulsion strategies in response to variations in local flow intensity (Gao et al. 2025). Behavioural assessments of rheotaxis have demonstrated that the upstream movement tendency of juvenile hybrid sturgeon is strongly dependent on flow velocity; when subjected to flow velocities of 0.10, 0.30 and 0.50 m/s, individuals predominantly display upstream advancement, station‐holding behaviour and downstream retreat, respectively (Dan et al. 2008, 2011). Fish also exhibit explicit preferences for specific flow velocities. For instance, silver carp prefer flow speeds of approximately 0.2 m/s–0.3 m/s, within which their passage efficiency and behavioural stability are maximised; beyond this optimal range, stress responses emerge and swimming performance declines (Li et al. 2025). Turbulence intensity similarly exerts a pronounced influence on fish movement trajectories and behavioural modes, challenging the adaptive capacities of different species and often manifesting as path deviation, disorientation or randomised swimming (Gao et al.). In some cases, the presence of localised micro‐turbulence within fishways can promote aggregation, particularly for small migratory fish that tend to congregate near turbulence–still–water interfaces (Turker and Kucukali 2025). Although carp typically display avoidance behaviour under high‐turbulence intensity (Mawer et al. 2023), lampreys may exhibit attraction to specific turbulence structures—such as low‐frequency shear zones—indicating that fish possess acute sensitivity to turbulence characteristics and respond selectively according to species‐specific ecological requirements (Dennis et al. 2025). Variations in flow conditions also carry substantial energetic implications, with gentle currents reducing energy expenditure and swift flow increasing metabolic cost (Brodersen et al. 2008). Nevertheless, the mechanisms by which flow regimes and hydraulic properties jointly shape movement patterns, hydraulic preference behaviours and energy utilisation—thereby modulating aggregation characteristics within fish schools—remain poorly elucidated.

Beyond hydraulic conditions, the visual environment also exerts a substantial influence on fish passage performance at dams (Ali et al. 2023). Fish possess the ability to perceive multiple visual attributes of surrounding objects, including brightness, hue and contours (Guthrie and Muntz 1986; Douglas and Hawryshyn 1990), and consequently exhibit diverse behavioural responses. For instance, approach behaviour refers to movement towards a visual stimulus, avoidance behaviour involves moving away from potentially threatening stimuli, whereas following behaviour describes the tendency to track and maintain proximity to moving objects or conspecifics (Rowland 1999; Xiang et al. 2022). Their spatial orientation capability is largely governed by the visual context in which they are embedded (Brown et al. 2010). Previous studies have demonstrated that fish often utilise nearby walls to maintain a stable upstream orientation, which may enhance their perception of flow direction. In addition to visual cues, this behaviour may also be influenced by the hydrodynamic properties of the boundary layer near channel walls, such as reduced velocity and modified turbulence structures (Bano et al. 2018; Gisen et al. 2022; Mozzi et al. 2025). Moreover, target objects present in the visual environment can function as guidance cues for individuals within a school. Empirical observations have shown that fish tend to congregate in areas containing duckweed or floating vegetation, a behaviour often associated with shelter seeking, as these structures can provide refuge and reduce perceived predation risk (Midwood and Chow‐Fraser 2010). Further research indicates that inter‐individual distance and visual angle serve as key determinants of school structure, with fish modulating their spacing based on the perceived angular size of neighbouring visual images (Wang et al. 2015; Yi et al. 2023; Simmons et al. 2026). Collectively, these findings highlight that visual orientation and guidance behaviours form a fundamental basis for schooling organisation and coordinated movement.

Although considerable progress has been made in characterising the macroscopic features of fish school morphology and group size, the mechanisms by which schooling behaviour responds to the combined influence of hydraulic conditions and visual environments remain relatively underexplored. To address this knowledge gap and to elucidate schooling intensity under hydraulic stimuli—specifically varying flow velocities and turbulence energy—as well as visual cues, such as wall orientation and obstacle guidance, the present study selected juvenile silver carp, a representative member of China's four major carps, as the focal species. Controlled behavioural experiments were conducted to document upstream movements within designated flow fields, enabling an evaluation of how fish respond to environmental factors, such as flow velocity, turbulence energy and the presence of walls and obstacles. Behavioural responses were quantified using indicators, including energy expenditure and schooling intensity. Furthermore, the study examined key behavioural traits (Tibing and Shuangke 2009; Khan 2006; Pettersson and Brönmark 1999), with particular emphasis on clarifying the decision‐making mechanisms elicited by hydraulic and visual stimuli, thereby aiming to quantify the relationships among hydraulic properties, visual perception and schooling intensity.

## 2 Materials and Methods

### 2.1 Subject

Silver carp, one of the four major Chinese carps, is a representative migratory species exhibiting typical riverine migration behaviour. The juvenile silver carp used in this study were obtained from a fish farm in Yidu, Hubei Province, with body lengths of 13.32 ± 1.58 cm and body weights of 34.75 ± 12.45 g. The fish were transported to the Ecological Hydraulic Laboratory of China Three Gorges University in oxygen‐enriched transport bags and subsequently held in a constant‐temperature laboratory rearing system to maintain optimal health and activity. The rearing water consisted of aerated tap water maintained at 22.2°C, with dissolved oxygen levels above 8 mg/L and a pH between 7.1 and 7.4; approximately one‐tenth of the total water volume was replaced daily. After acclimation, healthy and vigorous individuals were randomly selected for the release experiments. To ensure experimental rigour and avoid behavioural interference from repeated testing, each fish was used only once; after each trial, the fish were removed and returned to the rearing system for temporary holding.

### 2.2 Experimental Device

**FIGURE 1:**
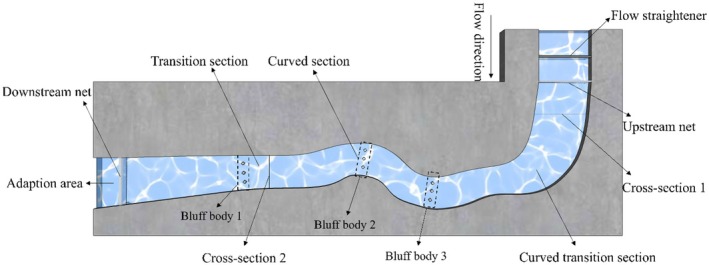
Schematic diagram of the experimental device.

To investigate the influence of schooling on upstream swimming against the current, experiments were conducted in an open, S‐shaped, curved recirculating flume (Figure 1). The facility was located in the Hydraulic Hall of China Three Gorges University and featured a maximum channel width of 2 m and a minimum width of 1.2 m. The radius of curvature in the bend section ranged from 0.13 to 0.37 m, with an overall bed slope of less than 6%. A flow‐straightening grid, an upstream fish‐blocking net, a flow‐conditioning baffle and a downstream net were sequentially installed along the flow direction. The flume consisted of a rectification zone, a curved transition section, a curved section, a straight transition section and an acclimation zone, enabling the replication of non‐uniform flow conditions comparable to those encountered in natural river environments. To eliminate external visual interference, both sides of the flume were covered with black tarpaulins.

The overall design followed the principles of low‐velocity, low‐turbulence and non‐uniform flow, ensuring that the experimental fish remained stable and avoided fatigue throughout their movement. Water was supplied from the underground recirculating reservoir of the Hydraulic Hall, with the temperature maintained at a constant level consistent with the fish rearing environment. Hydraulic conditions were adjusted by regulating the number of open valves. All experiments were conducted during daytime under indoor natural lighting, with ambient illuminance maintained between 40 lx and 120 lx. The flume was partitioned into six segments, each equipped with an infrared camera (DS‐2CD3T35D‐I; Hikvision) mounted 4.5 m above the ground to record the upstream swimming behaviour of the fish continuously.

### 2.3 Experimental Method

In this study, juvenile silver carp were selected as the experimental subjects. The water temperature set for the experiment was 22°C. Their perceptible flow velocity ranges from 0.07 to 0.13 m/s, and their critical swimming speed reaches 0.79 m/s. To ensure that the hydraulic conditions within the flume remained within the perceptual range of the fish while not exceeding their critical swimming capacity, three flow velocity intervals were established. The reported values represent the bulk (cross‐section averaged) flow velocity in the experimental flume and were measured using a flow velocity meter (LS300‐A; Nanjing Lanlu Electronics Co. Ltd., China). The selected velocity ranges were 0.30–0.50 m/s, 0.35–0.60 m/s and 0.40–0.70 m/s. A square columnar baffle was installed in the experimental section to generate a localised obstructed flow field, thereby simulating obstacle‐induced hydraulic conditions commonly encountered in natural rivers. For each of the three flow velocity regimes, groups of five fish were used as the schooling treatment units, and each treatment was replicated 10 times to ensure the reliability and repeatability of the experimental results.

Prior to the formal experiments, the flow velocity in the test area was adjusted and measured using a portable flow velocity meter (model LS300‐A; Nanjing Lanlu Electronics Co. Ltd., Nanjing, China) to ensure that the hydraulic conditions matched the prescribed experimental settings. Healthy and active individuals were then randomly selected and transferred to the flow‐adaptation zone for a 30‐min acclimation period. Following acclimation, the downstream net was lifted and all cameras were simultaneously activated to record the upstream swimming behaviour of the test fish continuously. Preliminary observations indicated that the average duration of upstream movement was 115.3 ± 66 s; therefore, each trial lasted up to 5 min. A fish was considered to have completed the upstream passage if it reached the upstream net area within this period. The trial was terminated earlier if all experimental fish arrived at the upstream area before the end of the observation period. Upon completion of each trial, video recording was terminated and the morphological parameters of the tested fish—including total length, body length and body weight—were measured and documented.

### 2.4 Hydraulic Calculation

The RNG *k–ε* turbulence model offers high computational efficiency, numerical stability and reliable accuracy in practical hydraulic simulations; thus, it was selected for the numerical analysis conducted in this study. Numerical simulations of the actual flow field were performed using Flow‐3D (v12.0), based on the hydrodynamic governing equations, including the continuity equation and the Reynolds‐averaged momentum equations. The relevant governing equations are as follows:

Continuity Equation (Tibing and Shuangke 2009):

(1) $$\frac{\partial \rho }{\partial t}+\frac{\partial \rho u_{i}}{\partial x_{i}}=0$$

Momentum Equation:

(2) $$\frac{\partial \mathrm{\rho u}}{\partial t}+\frac{\partial }{\partial x_{i}}\left(\rho u_{i}u_{j}\right)=-\frac{\partial p}{\partial x_{i}}+\frac{\partial }{\partial x_{i}}\left[\left(\mu +\mu_{t}\right)\left(\frac{\partial u_{i}}{\partial u_{j}}+\frac{\partial u_{j}}{\partial u_{i}}\right)\right]$$

*k* equation:

(3) $$\frac{\partial \left(\rho \varepsilon \right)}{\partial t}+\frac{\partial \left(\rho u_{i}k\right)}{\partial x_{i}}=\frac{\partial }{\partial x_{i}}\left[\left(\mu +\frac{\mu_{t}}{\sigma_{k}}\right)\frac{\partial k}{\partial x_{i}}\right]+G_{K}-\rho \varepsilon$$

*ε* equation:

(4) $$\frac{\partial \left(\rho \varepsilon \right)}{\partial t}+\frac{\partial \left(\rho u_{i}\varepsilon \right)}{\partial x_{i}}=\frac{\partial }{\partial x_{i}}\left[\left(\mu +\frac{\mu_{t}}{\sigma_{k}}\right)\frac{\partial \varepsilon }{\partial x_{i}}\right]+\frac{C_{1\varepsilon }^{*}\varepsilon }{k}G_{K}-C_{2\varepsilon }^{*}\rho \frac{\varepsilon^{2}}{k}$$

in the equations, t is time (s); u_i_ and u_j_ are the average fluid velocities in the u_i_ and u_j_ directions (m/s), respectively; ρ is the average water density (kg/m^3^); μ is the dynamic viscosity (N·s/m^2^); μ_t_ is the turbulent viscosity (N·s/m^2^); p is the hydrostatic pressure (Pa); k is the turbulent kinetic energy (m^2^/s^2^); ε is the turbulent energy dissipation rate (kg·m^2^/s^3^); the turbulence constant *σ*_ε is set to 1.39; *G*_*k*_ is the production term of turbulent kinetic energy.

(5) $$Gk=\mathrm{\mu t}\left(\frac{\partial \mathrm{\mu i}}{\partial \mathrm{\mu j}}+\frac{\partial \mathrm{\mu j}}{\partial \mathrm{\mu i}}\right)\frac{\partial \mathrm{\mu i}}{\partial \mathrm{xj}}$$

(6) $$C_{1\varepsilon }^{*}=C_{1\varepsilon }-\frac{\eta \left(1-\eta /\eta 0\right)}{1+{\beta \eta }^{3}}$$

(7) $$C_{2\varepsilon }^{*}=C_{2\varepsilon }+\frac{C_{\mu }{\rho \eta }^{3}\left(1-\eta /\eta^{3}\right)}{1+{\beta \eta }^{3}}$$

The empirical coefficients are *C*_*1ε*_ = 1.42, *C*_*2ε*_ = 1.92, $\eta ={\left(2S_{\mathrm{ij}}S_{\mathrm{ji}}\right)}^{0.5}\frac{k}{\varepsilon }$, *S*_*ij*_ is the norm of the strain rate tenso, $S_{\mathrm{ij}}=\frac{1}{2}\left(\frac{\partial \mu_{i}}{\partial x_{j}}+\frac{\partial \mu_{j}}{\partial x_{i}}\right)$ *η*_*0*_ is the typical value of *η* in uniform shear flow, *η0* = 4.38, *β* = 0.012, *i*、*j* = 1,2,3.

The Tru‐VOF method (Hirt and Nichols 1981) was used to solve the volume fraction continuity equation for the water–air two‐phase flow, thereby determining the exact position of the free water surface in the experimental flume. The fluid volume fraction is represented by *α*_*w*_, where *α*_*w*_ = 0 indicates that the computational cell contains only gas, and *α*_*w*_ = 1 indicates that the cell contains only fluid. The equation is as follows:

(8) $$\frac{\partial \alpha_{w}}{\partial t}+u_{i}\frac{\partial \alpha_{w}}{\partial x_{i}}=0$$

in the equation, t is time, and *u*_*i*_ represents the time‐averaged velocity component in each direction.

Two representative validation sections were selected to assess the accuracy of the simulated flow field. Velocity measurements were taken along each section at 0.1‐m intervals across the channel width, and the measured values were compared with the corresponding simulated velocities extracted from the numerical model at the same locations. The layouts of Sections 1 and 2 are shown in Figure 1.

To quantitatively evaluate the agreement between the measured and simulated velocities, the root mean square error (RMSE) was calculated as follows:

(9) $$\text{RMSE}=\sqrt{\frac{1}{n}\sum_{i=1}^{n}{\left(V_{s,j}-V_{m,i}\right)}^{2}}$$

where $V_{s,i}$ and $V_{m,i}$ denote the simulated and measured velocities at the i‐th point, respectively, and n is the number of measurement points.

**FIGURE 2:**
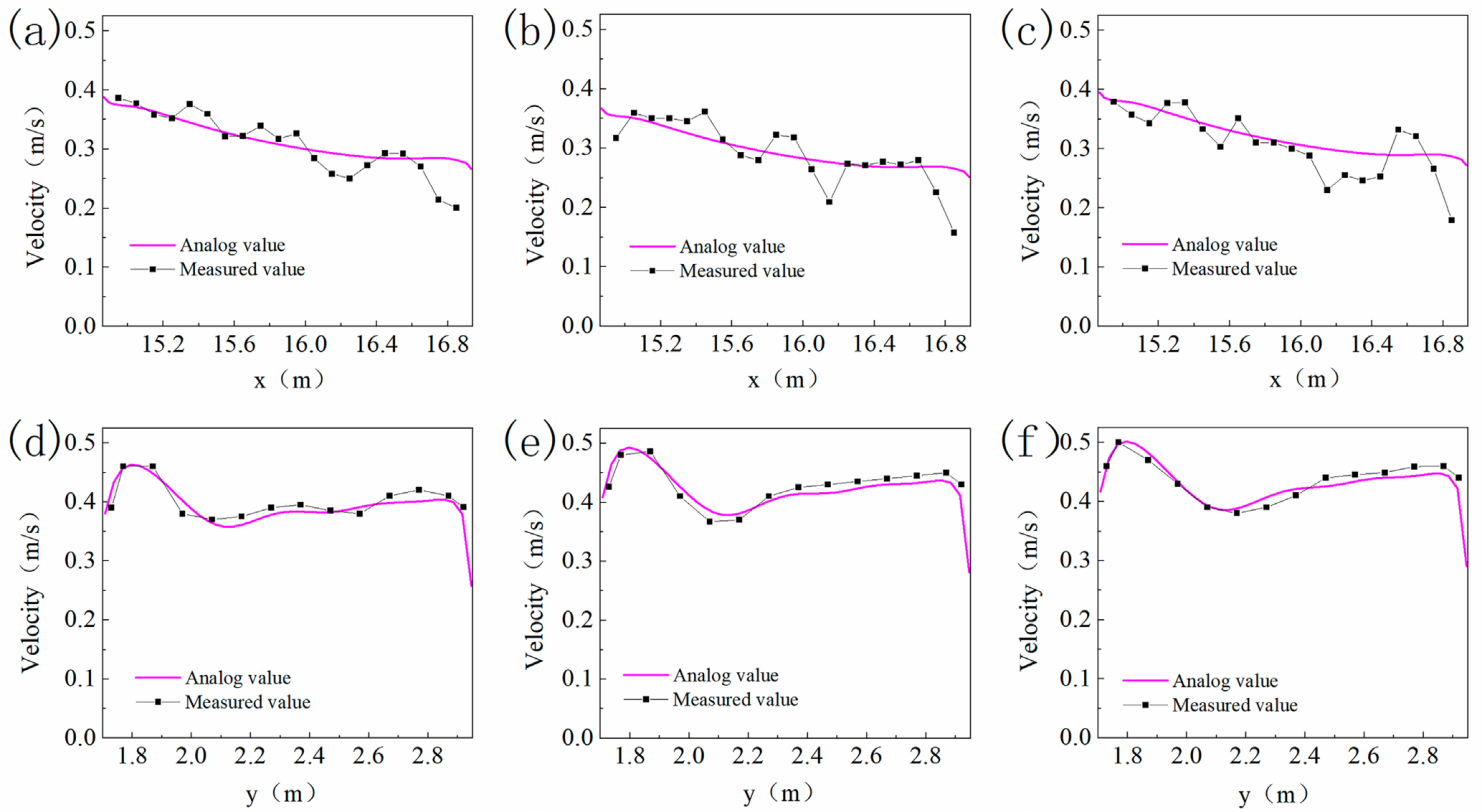
Cross‐section verification under different flow velocity conditions. (a)~(c) Verification of uniformity of inlet flow conditions, Section 1, comparison of flow velocity along the x‐direction of the flume. (d)~(f) Local obstructed flow field, Section 2, comparison of flow velocity along the y‐direction of the flume.

The comparisons under the three inflow velocity conditions showed that (Figure 2) at Section 1, the velocity gradually decreased from the left bank towards the right bank, whereas at Section 2, the velocity was relatively low in the mid‐right region, higher near the right bank and more uniform along the left bank. The simulated results reproduced these distribution characteristics well. The estimated RMSE values ranged from 0.01 to 0.05 m/s, with lower errors observed at Section 2 than at Section 1. This quantitative validation indicates that the numerical model can reasonably reproduce the measured flow field and is suitable for subsequent hydraulic analysis.

### 2.5 Data Analysis

Fish movements were recorded using a video camera mounted above the experimental flume to capture the entire observation area. The videos were recorded at 1280 × 720 pixels and 60 fps. The recorded videos were analysed using the open‐source video‐tracking software Tracker (Open Source Physics project). Individual fish positions were manually identified frame by frame, allowing the trajectories of the five fish within the flume to be reconstructed for subsequent behavioural analysis. The selected behavioural indicators were the influence of wall effects, upstream energy expenditure and schooling intensity.

#### 2.5.1 Hydraulic Influence

By overlaying flow‐field data with fish movement coordinates, the flow velocity and turbulence kinetic energy experienced by the fish at each moment during upstream swimming were obtained, enabling quantitative analysis of the instantaneous hydrodynamic environment. This approach provides an approximation of the hydrodynamic conditions encountered by the fish, whereas potential modifications of the local flow field caused by schooling behaviour were not explicitly considered.

#### 2.5.2 Upstream Energy Expenditure

The swimming resistance *f* of the fish body is given by (Khan 2006):

(10) $$f=0.5C_{d}\rho A_{s}{\left(U_{w}-U_{f}\right)}^{2}$$

in the equation, *C*_*d*_ is the drag coefficient, calculated using the following formula (Pettersson and Brönmark 1999): $C_{d}\approx 1.2C_{f}$,Cf=0.074Ref−0.2, In the equation, Ref=ρufLf/μ is the Reynolds number of the fish body (Webb 1971); ρis the density of the experimental water; $A_{s}=\alpha L_{f}^{\beta }$ is the wetted surface area of the fish body, *L*_*f*_ is the body length of the fish. Here, α and β are empirical coefficients, α=0.456, β=2.11. $U_{W}$ and $U_{f}$ represent the flow velocity and the swimming velocity of the experimental fish, respectively.

The absolute upstream trajectory length *S* is given by:

(11) $$S=|U_{w}-U_{f}|t$$

therefore, the cumulative upstream energy expenditure *E* is given by:

(12) $$E=\int_{0}^{S}|f|\mathrm{dS}$$

based on Equation (11), three metrics were defined to characterise energy expenditure at different temporal scales during upstream swimming. Instantaneous energy expenditure is the energy an individual fish consumes at a given moment to overcome water resistance, maintain body posture and generate propulsion. The 10‐s energy expenditure is the total energy consumed by an individual fish over the preceding 10‐s period before a given moment, reflecting short‐term energetic investment during sustained swimming. Cumulative energy expenditure represents the total energy consumed by an individual fish from the beginning of observation to a given time point (e.g., from the start of upstream swimming to the current moment). This multi‐scale energy framework allows the energetic consequences of fish movement to be examined at instantaneous, short‐term and cumulative levels.

#### 2.5.3 Visual Influence

##### 2.5.3.1 The Influence of the Side Wall Effect

Walls are an important environmental factor influencing fish behaviour, capable of exerting attraction, repulsion and constraint on surrounding fish (Leem et al. 2012), and may also affect interactions among individuals within a school (Mozzi et al. 2025). Experimental videos reflecting the upstream trajectories of fish were randomly selected to determine the shortest distance between the centre of the fish's body and the wall at each time point, as well as the change in this distance calculated by subtracting the previous time point from the current one. By examining the relationship between these variables, the constraining influence of physical walls on upstream orientation can be assessed.

##### 2.5.3.2 Obstacle‐Guidance Interaction Intensity

The visual perception of the target fish is an important factor influencing its movement orientation and trajectory prediction (Rodríguez et al. 2021). This study considers guidance as the dominant mechanism of fish interactions, positing that changes in the swimming behaviour of all neighbouring individuals within the target fish's visual field can exert a pulling effect on the target fish, whether attractive or repulsive. This guidance interaction is referred to as the guidance motion vector. The guidance motion vector $\vec{v_{B}}$ can be obtained by calculating the relative motion between the target fish A and the obstacle B. The direction of the target fish's relative motion vector to the obstacle $\vec{v_{\mathrm{AB}}}$ determines the direction of the guidance vector $\vec{v_{B}}$ for the next time step. The magnitude of $\vec{v_{B}}$ is influenced by the azimuth angle θ of the obstacle relative to the head orientation of the target fish and the distance d between the target fish and the obstacle. The calculation formula is as follows (Lin 2023):

(13) $$\vec{v_{B}}=f_{g}\left(\theta ,d\right)+\vec{R_{g}}$$

in the equation, $\vec{R_{g}}$ is a random vector in the same direction as $\vec{v_{B}}$ representing possible random fluctuations of the guidance vector. Meanwhile, the guidance vector can also be calculated as follows:

(14) $$\vec{v_{B}}={\vec{V_{\mathrm{AB}}}}^{*}d_{g}$$

in the equation, $d_{g}$ presents the magnitude of the guidance vector's influence, $\vec{v_{B}}$ which can be regarded as the projection of the target fish's acceleration vector onto $\vec{v_{\mathrm{AB}}}$, the additional velocity component of the target fish's motion along its relative motion vector when influenced by the guidance effect.

**FIGURE 3:**
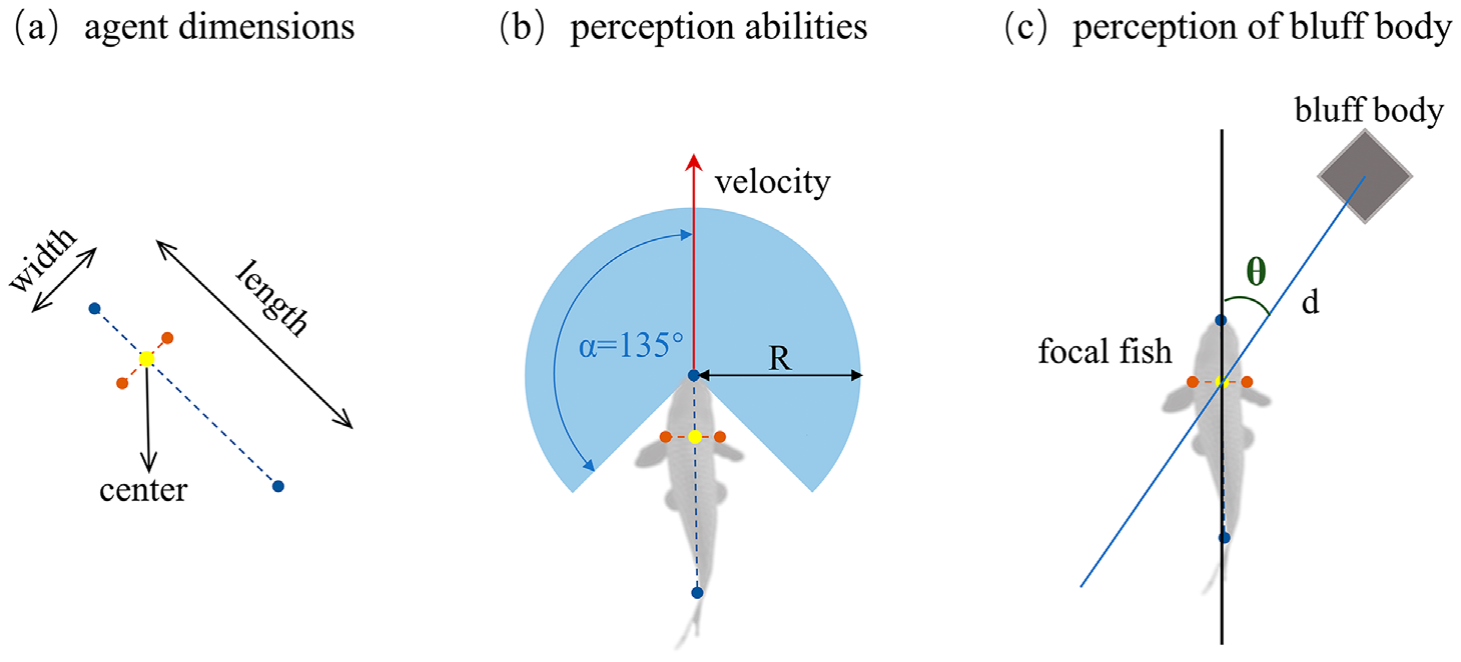
Schematic diagram of simulating fish structure and its perception ability. (a) Fish body morphology; (b) fish sensory range; (c) directional perception of the flow obstacle—schematic diagram illustrating the definition of kinematic variables used in the trajectory analysis.

The schematic diagram is as follows (Figure 3).

#### 2.5.4 Schooling Intensity

The interactions among individuals within a fish school have always been a crucial aspect in analysing schooling behavioural activities (de Lamo et al. 2025; Leem et al. 2012). For each of the five fish in the experimental group, an individual interaction analysis was conducted, with the fish exerting influence as the neighbour fish and the fish being influenced as the focal fish. For the five fish in each experimental group, interaction analyses were conducted by treating each fish as the focal individual in turn, whereas the remaining fish were considered neighbouring individuals influencing its movement. The perceptual zone of the focal fish was divided, from near to far, into a repulsive‐guidance zone, a guidance zone and an attractive‐guidance zone. Relevant studies have shown that the radii of the repulsive‐guidance zone, guidance zone and attractive‐guidance zone for juvenile silver carp are 0~0.11 m, 0.11~0.15 m and 0.15~0.38 m, respectively (Y, 2023). When beyond this range, the neighbour fish are considered unable to influence the focal fish. Based on this, the present study characterises the magnitude of schooling intensity. At each time point, the distance between the focal fish and each neighbouring fish is calculated, and each neighbour is classified according to its position within the focal fish's guidance domains, along with the number of neighbours in each domain. The three domains, from near to far, are assigned intensity weights of 3, 2 and 1, respectively. The schooling intensity SS is defined as:

(15) $$\mathrm{SS}=3N_{1}+2N_{2}+N_{1}$$

in the equation, *SS* represents the schooling intensity, and *N*_*1*_, *N*_*2*_ and *N*_*3*_ correspond to the numbers of neighbour fish located in the focal fish's repulsive‐guidance zone, guidance zone and attractive‐guidance zone, respectively. The weighting scheme reflects the relative behavioural influence of neighbouring fish at different interaction distances, with higher weights assigned to short‐range interactions **t**o reflect the spatial density of fish within the school.

## 3 Results

### 3.1 Analysis of Flow‐Field Simulation Results

**FIGURE 4:**
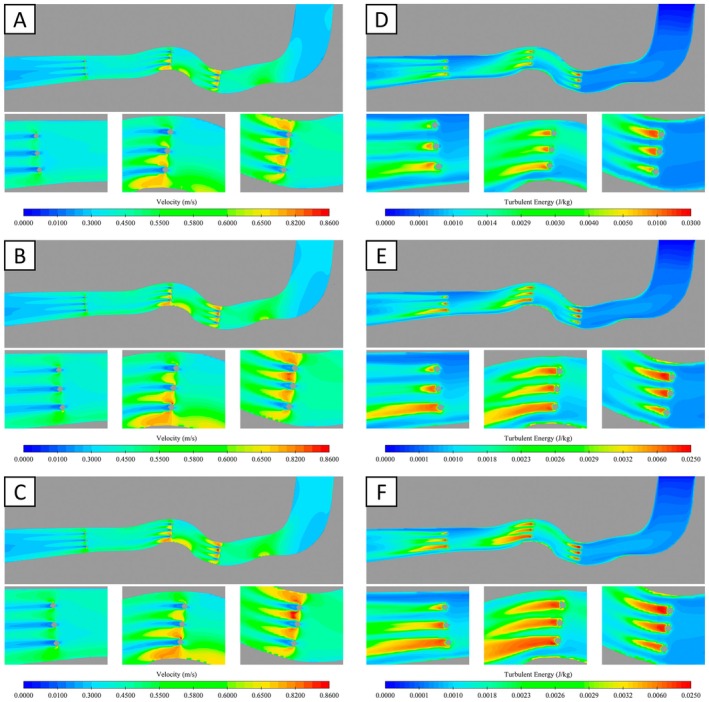
Numerical simulation results under different flow conditions (A)~(C) Flow velocity distributions under Condition 1, Condition 2 and Condition 3, respectively. (D)~(F) Turbulence kinetic energy distributions under Condition 1, Condition 2 and Condition 3, respectively.

The numerical simulation results revealed the spatial distributions of flow velocity and turbulence kinetic energy (Figure 4). From the upstream region to the zone immediately preceding the first row of obstacles, both flow velocity and turbulence kinetic energy remained relatively uniform, with pronounced velocity gradients occurring only within sections of substantial channel curvature. In the middle and downstream regions, the combined effects of obstacles and curved channel segments produced considerable heterogeneity in hydraulic parameters, which gradually diminished as the flow entered the straight transition section. Accelerated flow developed in the constricted spaces between obstacles, leading to elevated velocities, whereas pronounced velocity reductions occurred immediately downstream of the obstacles. The highest velocities were observed near the point of maximum curvature adjacent to the obstacles. Turbulence intensity varied progressively between obstacles; however, turbulence kinetic energy increased sharply in the wake regions downstream of the obstacles and decayed with distance, with influence zones biased towards the concave bank. Peak turbulence kinetic energy consistently occurred directly behind the obstacles. Additionally, increases in the imposed flow velocity resulted in higher maximum velocities and greater turbulence kinetic energy, accompanied by an expansion of the high‐velocity and high‐turbulence regions.

**FIGURE 5:**
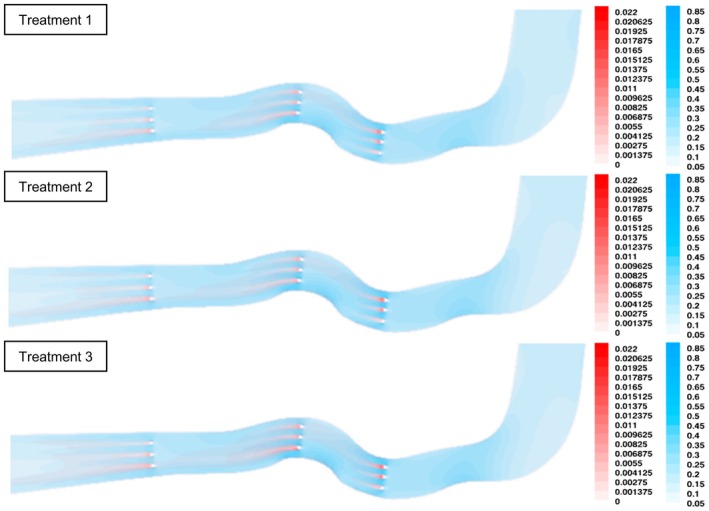
Superposition diagram of turbulent velocity In the figure, red represents the turbulent kinetic energy (J/kg), and blue represents the flow velocity (m/s).

To further clarify the hydraulic structure, processed velocity fields and turbulence kinetic energy distributions were superimposed (Figure 5), with blue indicating flow velocity magnitude and red representing turbulence kinetic energy. Under all three hydraulic conditions, turbulence kinetic energy remained low in the upstream region, increasing slightly in curved sections. Flow velocity exhibited a clear lateral asymmetry, with higher velocities near the convex bank than the concave bank. In the vicinity of obstacles, high‐velocity jets formed between obstacles, whereas low‐velocity, high‐turbulence zones developed immediately downstream of each obstacle. The turbulence kinetic energy fields displayed elongated, strip‐like patterns aligned with the primary flow direction, and the spatial extent of these structures increased progressively with increasing flow velocity.

### 3.2 The Constraint Effect of the Underwater Side Wall on Fish

**FIGURE 6:**
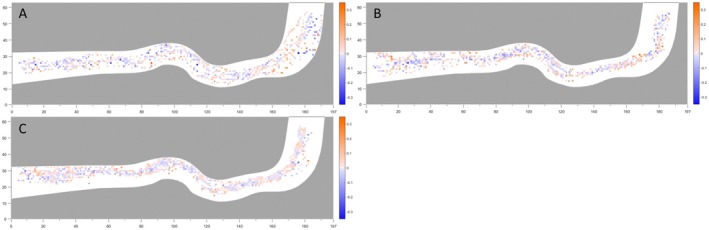
Superposition of juvenile silver carp distribution and distance variation (A)~(C) Distribution of changes in distance from the wall for a single fish under Flow Conditions 1, 2 and 3, respectively. In the figure, the orange colour represents the distance moving away from the wall (m), and the blue colour represents the distance moving towards the wall (m).

The spatial distribution of the experimental fish within the flume, together with their distance from the channel walls, was superimposed and analysed to elucidate movement trends associated with wall effects (Figure 6). Under all three flow conditions, the variability in the fish–wall distance was markedly greater in the central region of the flume, whereas distance fluctuations became progressively smaller as the fish approached the channel boundaries. Furthermore, as flow intensity increased, the overall range of fish–wall distances within the flume decreased, indicating a contraction of lateral movement with increasing hydraulic forcing.

**FIGURE 7:**
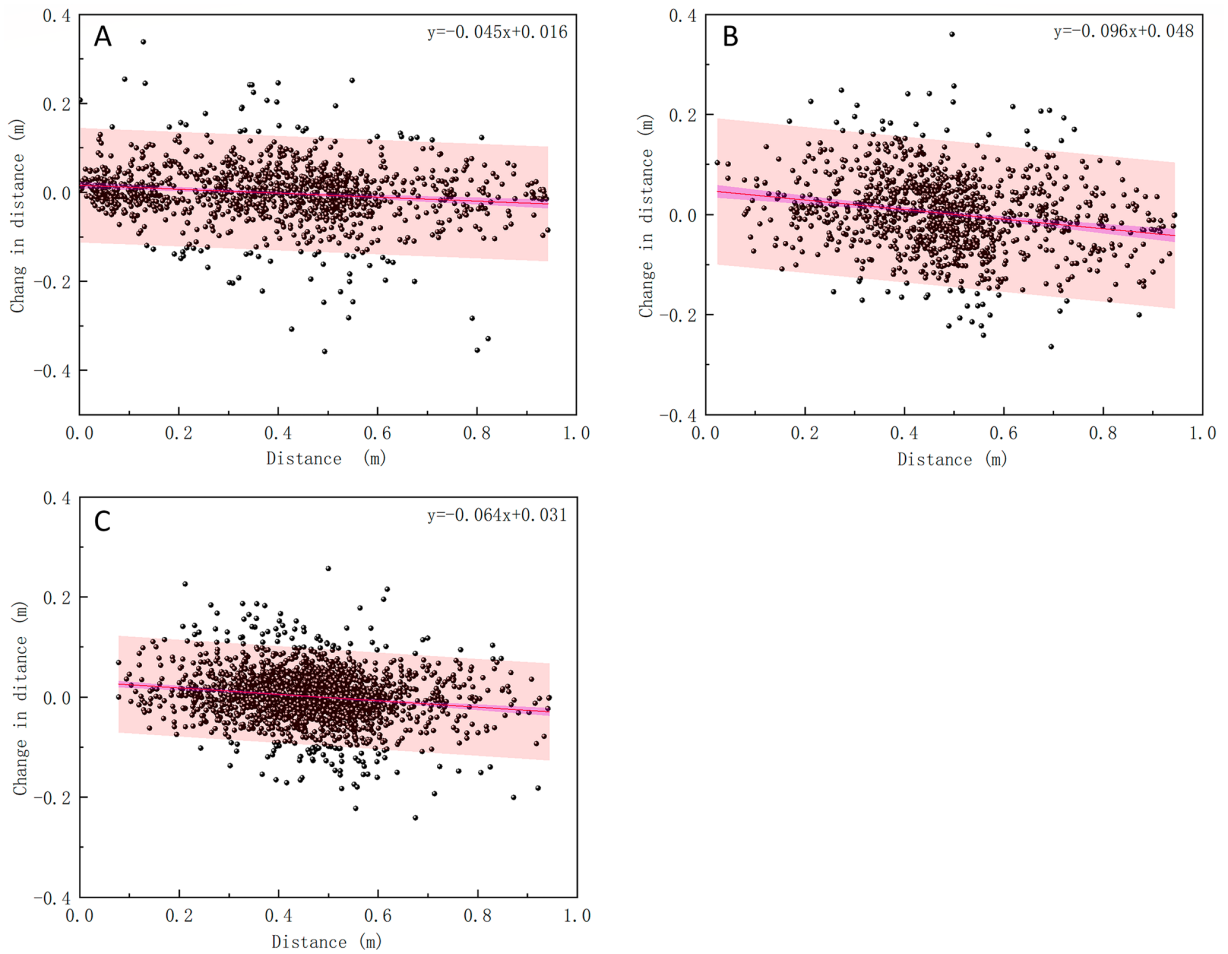
Relationship between distance from juvenile silver carp to boundary and distance variation (A)~(C) Relationship between the distance to the boundary and changes in distance from the wall for a single fish under Flow Conditions 1, 2 and 3, respectively.

By analysing the movement behaviour of juvenile silver carp in proximity to the physical walls, the distance between the fish centroid and the wall, as well as its temporal variation, was quantified. The relationship between these variables was subsequently fitted to assess the constraining influence of physical walls on upstream orientation (Figure 7). Across all three flow conditions, fish generally exhibited a ‘near‐approach, far‐recede’ tendency with respect to the wall, tending to move away when very close to it and approach it when farther away. Under Flow Conditions 1 and 2, the distribution of distance‐change values was relatively scattered, indicating greater fluctuations in lateral movement. In contrast, under Flow Condition 3, the distance‐change values were more concentrated, with most variations remaining within approximately ±0.1 m. Across all flow regimes, the preferred wall‐distance range for juvenile silver carp was approximately 0.2–0.6 m, suggesting a stable behavioural preference for maintaining moderate proximity to the physical boundary.

**FIGURE 8:**
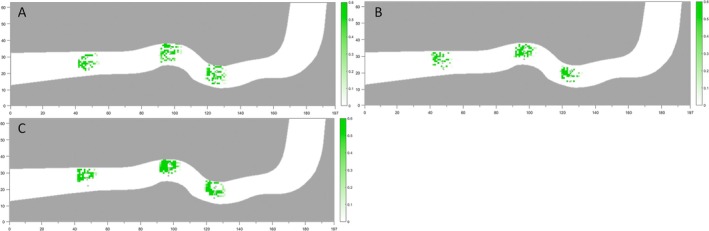
Guiding interaction effect of obstacles on silver carp juveniles (A)–(C) Distribution of the guidance interaction effect from obstacles on a single fish under Flow Conditions 1, 2 and 3, respectively.

The spatial distribution of the experimental fish within the flume was superimposed with the quantified degrees of guidance interaction relative to the obstacles to evaluate the intensity of obstacle‐induced behavioural guidance (Figure 8). The results showed that the influence of obstacle guidance was strongest within approximately 0.5 m of the obstacle, displaying a clear ‘near‐strong, far‐weak’ pattern. Moreover, the guidance effect was more pronounced when the obstacle was positioned upstream of the target fish than when it was located downstream. When the obstacle was situated directly behind the fish, particularly within an angular range of approximately 30°, the interaction intensity was negligible, indicating minimal behavioural influence from the obstacle in this downstream sector.

### 3.3 Upstream Energy Expenditure Under Different Experimental Conditions

#### 3.3.1 Instantaneous Energy Expenditure Distribution

**FIGURE 9:**
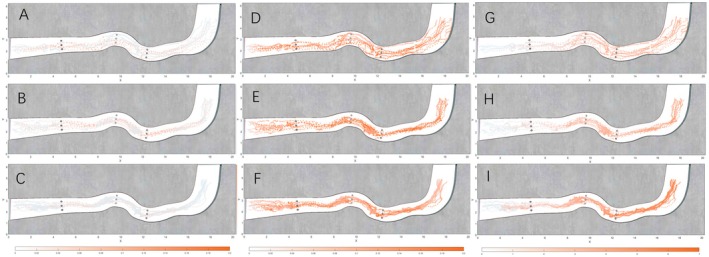
Instantaneous energy consumption distribution map of silver carp juveniles (A) Instantaneous energy expenditure distribution of experimental fish under Flow Condition 1. (B) Instantaneous energy expenditure distribution under Flow Condition 2. (C) Instantaneous energy expenditure distribution under Flow Condition 3. (D) 10‐s energy expenditure distribution under Flow Condition 1. (E) 10‐s energy expenditure distribution under Flow Condition 2. (F) 10‐s energy expenditure distribution under Flow Condition 3; (G) Cumulative energy expenditure distribution under Flow Condition 1. (H) Cumulative energy expenditure distribution under Flow Condition 2. (I) Cumulative energy expenditure distribution under Flow Condition 3.

The spatial pattern of instantaneous energy expenditure during upstream swimming was determined by mapping the instantaneous energy values onto the corresponding upstream trajectory points of juvenile *Hypophthalmichthys molitrix* (Figure 9A–C). Under low‐flow conditions, the upstream movement paths were relatively dispersed, with individuals exhibiting diverse swimming routes throughout the flume. Instantaneous energy expenditure was also broadly distributed, with no clear or persistent area of concentration across trials. Under medium‐flow conditions, trajectory points in the downstream region were more scattered, whereas upstream paths tended to become more convergent as individuals progressed upstream. Instantaneous energy expenditure was higher in the wake regions downstream of the obstacles and lower farther away. Under high‐flow conditions, the upstream trajectories of the experimental fish exhibited a narrower and more concentrated distribution, indicating more consistent path selection. Notably, the instantaneous energy expenditure in the vicinity of Obstacles 1 and 3 was substantially higher than in other regions, highlighting the intensified energetic cost imposed by localised hydraulic disturbances at elevated flow velocities.

#### 3.3.2 10‐s Energy Expenditure Distribution

To characterise short‐term energy use during upstream swimming, the 10‐s energy expenditure values were assigned to the corresponding trajectory points of juvenile *Hypophthalmichthys molitrix* , thereby revealing their spatial distribution. These values represent the cumulative instantaneous energy expenditure over the 10 s preceding each trajectory position (Figure 9D–F). Under low‐flow conditions, the 10‐s energy expenditure was markedly higher in the downstream region of Obstacle 2 and immediately upstream of Obstacle 3. Overall, energy expenditure exhibited a clear longitudinal pattern, with substantially higher 10‐s expenditure in the downstream portion of the flume relative to the upstream region. Under medium‐flow conditions, a similar trend was observed: the 10‐s energy expenditure increased sharply immediately upstream of Obstacle 3, with slightly elevated values also present near Obstacle 1, whereas energy expenditure in the remaining regions remained relatively uniform. Under high‐flow conditions, the spatial distribution of elevated 10‐s energy expenditure closely resembled that observed under low‐ and medium‐flow conditions, with the downstream region consistently exhibiting higher values and the upstream region maintaining comparatively lower energetic expenditure.

#### 3.3.3 Cumulative Energy Expenditure Distribution

The spatial distribution of cumulative energy expenditure during upstream movement was obtained by linking each trajectory point to the total energy consumed by juvenile *Hypophthalmichthys molitrix* up to that position. Here, cumulative energy expenditure refers to the sum of instantaneous energy expenditure from the start of swimming to the given trajectory point (Figure 9G–I). Under low‐flow conditions, cumulative energy expenditure increased progressively with upstream distance, with the rate of increase moderating after the fish passed through the curved section. The maximum cumulative energy expenditure recorded for the group was 4.03 J. Under medium‐flow conditions, a similar trend was observed: cumulative energy expenditure rose substantially as the fish traversed the curved section near Obstacle 3, with overall values slightly exceeding those recorded under low‐flow conditions. The maximum cumulative energy expenditure for this group reached 4.67 J. Under high‐flow conditions, some individuals exhibited an abrupt increase in cumulative energy expenditure upon entering the upstream flow region from the downstream section. Moreover, cumulative energy expenditure at comparable upstream positions was significantly greater than in the low‐ and medium‐flow scenarios, with a maximum of 6.67 J.

### 3.4 School Intensity

**FIGURE 10:**
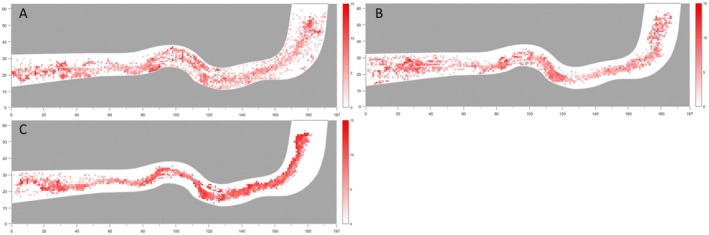
School strength distribution map of silver carp juveniles (A)~(C) Flow velocity distributions under Flow Conditions 1, 2 and 3, respectively. (D)~(F) Turbulence kinetic energy distributions under Flow Conditions 1, 2 and 3, respectively. In the figure, red represents the school strength.

By superimposing the schooling intensity values with the spatial distribution of experimental fish within the flume, the overall spatial pattern of schooling intensity during upstream movement was obtained (Figure 10). Under low‐flow conditions, the fish were relatively dispersed, with several individuals exhibiting wall‐effect behaviour during upstream progression. Regions of elevated schooling intensity were mainly concentrated in the downstream straight transition section, at both the entrance and exit of the middle curved section, and near the upstream curved transition section. Under medium‐flow conditions, the distribution of fish became more aggregated, accompanied by a reduction in wall‐effect behaviour. The locations of higher schooling intensity were generally consistent with those observed under low‐flow conditions. Under high‐flow conditions, fish aggregation was most pronounced, with wall‐effect behaviour nearly absent and upstream movement occurring predominantly along the main flow axis. Correspondingly, schooling intensity was substantially higher than under both low‐ and medium‐flow conditions.

### 3.5 The Schooling Intensity is Affected by Many Factors Such as Flow Velocity, Turbulent Kinetic Energy, Energy Consumption and Vision

Using multiple linear regression analysis (Table 1), we further examined the comprehensive effects of flow velocity, turbulence kinetic energy, instantaneous energy expenditure, 10‐s energy expenditure, cumulative energy expenditure, wall‐effect intensity and obstacle‐guidance interaction intensity on the schooling intensity of juvenile *Hypophthalmichthys molitrix* . The results revealed the following:

1. The variance inflation factor (VIF) values for flow velocity, turbulence kinetic energy, instantaneous energy expenditure, 10‐s energy expenditure, cumulative energy expenditure, wall effect and obstacle‐guidance interaction intensity were all close to 1, indicating that no substantial multicollinearity existed among the independent variables.
2. Flow velocity, instantaneous energy expenditure, obstacle‐guidance interaction intensity and the interaction terms velocity × turbulence kinetic energy, velocity × instantaneous energy expenditure, turbulence kinetic energy × instantaneous energy expenditure and 10‐s × cumulative energy expenditure exerted measurable influences on schooling intensity. Among these factors, flow velocity, the interaction between flow velocity and turbulence kinetic energy, and the interaction between 10‐s and cumulative energy expenditure exhibited the most pronounced effects.

**TABLE 1:** Multiple linear regression analysis of the effects of flow velocity, turbulent kinetic energy, visual perception and three kinds of motion energy consumption on the strength of silver carp swarm.

Index	Fish school strength		
	VIF	*F*	*p*
Velocity	1.6841	7	0.0065
TKE	1.3943	3.59	0.1063
Instantaneous energy expenditure distribution	1.9717	18.54198996	0.0121
10‐s energy expenditure distribution	1.9730	0.03	0.5846
Cumulative energy expenditure distribution	1.3276	0.57	0.9982
Wall‐effect intensity	1.0346	2.2	0.7629
Obstacle‐guidance interaction intensity	1.0632	2.17	0.0407
Velocity × TKE	—	8.63	0.0007
Velocity × Instantaneous energy expenditure distribution	—	5.12	0.0423
Velocity × 10‐s energy expenditure distribution	—	0.15	0.5461
Velocity × Cumulative energy expenditure distribution	—	96.11	< 0.0001
Velocity × wall‐effect intensity	—	3.21	0.2939
Velocity × Obstacle‐guidance interaction intensity	—	1.87	0.0535
TKE × Instantaneous energy expenditure distribution	—	4.9	0.0217
TKE × 10‐s energy expenditure distribution	—	0.09	0.6806
TKE × Cumulative energy expenditure distribution	—	5.01	0.1167
TKE × wall‐effect intensity	—	1.05	0.6637
TKE × Obstacle‐guidance interaction intensity	—	1.23	0.5987
Instantaneous energy expenditure distribution×10‐s energy expenditure distribution	—	2.43	0.1203
Instantaneous energy expenditure distribution × Cumulative energy expenditure distribution	—	0.1	0.9511
Instantaneous energy expenditure distribution × wall‐effect intensity	—	2.21	0.3933
Instantaneous energy expenditure distribution × Obstacle‐guidance interaction intensity	—	1.82	0.3839
10‐s energy expenditure distribution×Cumulative energy expenditure distribution	—	7.72	0.0044
10‐s energy expenditure distribution × wall‐effect intensity	—	1.46	0.7734
10‐s energy expenditure distribution × Obstacle‐guidance interaction intensity	—	0.02	0.751
Cumulative energy expenditure distribution × wall‐effect intensity	—	1.51	0.2332
Cumulative energy expenditure distribution × Obstacle‐guidance interaction intensity	—	1.29	0.23163
Wall‐effect intensity × Obstacle‐guidance interaction intensity	—	0.01	0.6266

## 4 Discussion

### 4.1 Effects of Hydraulic Factors on Schooling Intensity During Upstream Migration

The results suggest that differences in flow velocity and turbulence conditions jointly influenced schooling intensity and upstream swimming behaviour in juvenile *Hypophthalmichthys molitrix* . Previous studies have shown that fish may exhibit different preferences for hydraulic conditions under different flow‐field settings, including variations in channel structure, boundary effects and local turbulence distributions. As a result, differences in experimental configurations may lead to variation in the preferred velocity ranges reported among studies. Notably, such hydraulic preferences may also be modulated by schooling behaviour—social interactions within groups can regulate individual responses to flow velocity, turbulence and other hydraulic factors, thereby shaping distribution patterns and movement strategies in complex flow fields. Related research has demonstrated that as group size increases, exploratory behaviour and swimming activity likewise intensify, although the magnitude of this enhancement is modulated by flow velocity (Mozzi, Nyqvist, et al. 2024). Conversely, increasing flow velocity can strengthen schooling effects, but only up to an upper threshold (Cao et al. 2025). For juvenile silver carp, previous observations in enclosed flume environments have identified a preferred velocity range of 0.15–0.19 m/s (Yuanjie 2021). In the present study, however, juvenile silver carp did not display a distinct velocity preference, possibly because the continuously changing flow direction during upstream migration altered their swimming strategy (Das et al. 2023; Porfiri et al. 2022). Rather than maintaining a stable preference for a specific local velocity, the fish appeared to respond to increasing flow velocity by strengthening aggregation and moving more consistently along the dominant flow direction.

In addition to flow velocity, the present study also showed that juvenile silver carp tended to ascend more rapidly through regions characterised by relatively high‐flow velocity and low‐turbulence kinetic energy, rather than exhibiting a distinct preference for a specific turbulence level. Previous research by Tan et al. reported that the preferred turbulence kinetic energy for juvenile silver carp ranges from 0.020 to 0.035 J/kg (Tan et al. 2018). In our experiments, no substantial differences in schooling intensity were observed within regions influenced solely by turbulence kinetic energy. However, in the high‐velocity, low‐turbulence zones adjacent to obstacles, schooling intensity decreased markedly, suggesting that variations in turbulence kinetic energy exert only a limited influence on schooling intensity under relatively mild flow conditions. Only in obstacle‐dominated regions—where elevated flow velocity co‐occurs with reduced turbulence—did the fish show a tendency to consistently select high‐velocity corridors as ‘efficient pathways’ for rapid upstream passage.

### 4.2 Effects of Energy Expenditure on Schooling Intensity During Upstream Migration

Schooling represents a fundamental counter‐current strategy in fish migration, enabling individuals to enhance upstream passage efficiency while substantially reducing energy consumption during long‐distance movement (Daghooghi and Borazjani 2015; Jiang et al. 2023; Tian‐Dong et al. 2021). Under complex hydraulic conditions, fish frequently adopt schooling behaviour to improve their ability to resist adverse flows (Xiaotao et al. 2024). Moreover, individuals within a school can achieve energy savings across a range of swimming speeds by exploiting the hydrodynamic benefits of group formation (Marras et al. 2015). In the present study, schooling intensity increased progressively with rising background flow velocity across all tested conditions, indicating that neither short‐term nor cumulative increases in energy expenditure alone triggered schooling behaviour. Instead, when fish encountered high‐velocity barriers, the associated rapid escalation in energetic demand promoted tighter group cohesion to maintain upstream progression. In addition, under low‐velocity, high‐turbulence conditions, the main flow's guiding effect was relatively weak, whereas local flow fluctuations were more pronounced. Under these conditions, fish were likely required to perform more frequent posture adjustments and movement corrections, making it more difficult to maintain stable relative positions within the group, explaining the marked decrease in the intensity of schooling. At moderate velocities, energy expenditure remained relatively low, whereas in the high‐velocity regions surrounding obstacles, energetic costs increased sharply. These patterns suggest that, after traversing velocity barriers, fish employed a short‐burst acceleration strategy within the lower‐velocity, lower‐turbulence zones downstream of obstacles to pass through quickly and avoid reforming dense schools. This behaviour suggests that, when physiological capacity permits, fish tend to reduce prolonged exposure to flow conditions associated with higher energetic cost.

### 4.3 Effects of the Visual Environment on Schooling Intensity During Upstream Migration

The visual environment exerts a non‐negligible influence on upstream fish movement. Visual obstacles such as channel walls and vegetation can alter local flow patterns, prompting fish to adopt avoidance strategies that subsequently affect schooling behaviour (Nguyen et al. 2016). Interactions among individuals are further shaped by their perceptual range and allocation of visual attention, both of which regulate swimming strategies and the coordination of group movement (Ito and Uchida 2024; Wang et al. 2022). In addition, the effect of visual cues is constrained by the fish's visual field. Numerous studies have demonstrated that cyprinids possess a monocular visual field of approximately 150°–170°, with a binocular overlap of about 30°–45° (Fitzgibbon 2020; Miles 2022), consistent with the behavioural patterns observed in this study. Our results showed that fish tended to maintain a preferred distance of 0.2–0.6 m from the channel walls and exhibited a boundary‐response pattern characterised by ‘It exhibits a visual‐based wall‐seeking behaviour, characterised by a tendency to approach the wall within a certain distance, and to move away when beyond a threshold distance’. This phenomenon suggests that, in addition to lateral‐line sensing of wall‐reflected flows, visual perception plays an important role in regulating fish–wall spacing. Furthermore, the markedly reduced schooling intensity downstream of obstacles indicates that visual occlusion generated by obstacles can disrupt directional judgement within the group. Such visual interference likely helps fish recognise and avoid upstream obstacles during rapid upstream movement. In summary, the hydrodynamic environment, energetic expenditure and visual conditions collectively constitute the primary drivers regulating schooling intensity. The findings indicate that schooling intensity is not static; rather, it represents a dynamic behavioural adjustment that fish make in response to flow stability, energetic cost and navigational or avoidance requirements. Fundamentally, this modulation reflects an adaptive strategy by which fish balance the need to conserve energy during long‐distance migration with the need to respond to immediate environmental risks.

### 4.4 Recommendations for Flow‐Field Optimisation of Fish Passage Facilities

Building on mechanistic insights into schooling‐based upstream movement, optimising fish passage facilities requires hydraulic and structural designs that actively support and reinforce natural schooling behaviour. This requires a systematic refinement of the internal flow regime through strategic placement of flow‐guiding structures and optimised pool geometry to suppress local turbulence and enhance overall flow stability, thereby mitigating the dispersive effects of low‐velocity, high‐turbulence zones and preserving school cohesion and movement continuity. Furthermore, establishing a spatial sequence with well‐defined velocity gradients is critical; providing distinct low‐velocity ‘energy refuge’ zones immediately downstream of high‐energy segments aligns with the behavioural strategy of incurring short‐term energetic costs for long‐term migratory gains, while offering hydraulic conditions conducive to energy recovery and group reorganisation. Careful planning of obstacle spatial arrangement is also essential: excessively complex structures along primary flow paths can amplify individual avoidance responses and disrupt group cohesion, whereas strategic utilisation of sidewall boundary effects can promote natural aggregation and enhance the consistency of upstream progression. Finally, greater emphasis should be placed on improving hydraulic continuity between fishways and natural river channels by engineering hydraulic alignment at key transition points, such as entrances and exits to create clear, directional flow pathways that effectively guide fish towards and into the primary upstream route. Collectively, these coordinated measures—aimed at simulating and reinforcing the hydraulic characteristics of natural rivers that promote schooling—establish a scientific foundation for enhancing the ecological performance and passage efficiency of fishway systems.

## Author Contributions

**Ding Haoyu:** conceptualization (equal), formal analysis (equal), methodology (lead), software (equal), validation (equal), writing – original draft (lead), writing – review and editing (equal). **Lin Chenyu:** conceptualization (equal), funding acquisition (lead), project administration (lead), supervision (equal), writing – original draft (equal), writing – review and editing (equal). **Wu Yujiao:** conceptualization (equal), supervision (equal). **Yang Zijing:** conceptualization (equal), data curation (equal). **Shi Xiaotao:** conceptualization (equal), funding acquisition (equal), project administration (equal). **Liang Zezhang:** conceptualization (equal), formal analysis (equal), software (equal). **Rong Guiwen:** conceptualization (equal), supervision (equal). **Xu Jiawei:** software (equal), supervision (equal). **Luo Jia:** project administration (equal), supervision (equal). **Yu Lixiong:** conceptualization (equal), supervision (equal).

## Funding

This work was supported by the Engineering Research Centre of Eco‐environment in Three Gorges Reservoir Region, Ministry of Education (2025KF02), the National Natural Science Foundation of China (32202942, 52279055, 52279069, 52509103 and W2511058) and the Natural Science Foundation of Hubei Province, China (2023AFA005 and 2024AFB292).

## Ethics Statement

This study adheres to the guidelines of the China Three Gorges University for the use of wild animals. Animals were fostered following the Guide to the Care and Use of Experimental Animals. No fish casualty occurred in this study. All procedures were approved by the Animal Protection Committee of China Three Gorges University.

## Conflicts of Interest

The authors declare no conflicts of interest.

## Supporting information


**Data S1:** ece373659‐sup‐0001‐Supinfo.zip

## Data Availability

All required data are provided as Supporting Information.
